# Blood Lead Concentrations < 10 μg/dL and Child Intelligence at 6 Years of Age

**DOI:** 10.1289/ehp.10424

**Published:** 2007-11-20

**Authors:** Todd A. Jusko, Charles R. Henderson, Bruce P. Lanphear, Deborah A. Cory-Slechta, Patrick J. Parsons, Richard L. Canfield

**Affiliations:** 1 Department of Epidemiology, School of Public Health and Community Medicine, University of Washington, Seattle, Washington, USA; 2 Department of Human Development, Cornell University, Ithaca, New York, USA; 3 Cincinnati Children’s Environmental Health Center, Cincinnati Children’s Hospital Medical Center, Cincinnati, Ohio, USA; 4 Department of Environmental Medicine, University of Rochester School of Medicine and Dentistry, Rochester, New York, USA; 5 Trace Elements Laboratory, Wadsworth Center, New York State Department of Health, Albany, New York, USA; 6 Department of Environmental Health Sciences, School of Public Health, The University at Albany, State University of New York, Albany, New York, USA; 7 Division of Nutritional Sciences, Cornell University, Ithaca, New York, USA

**Keywords:** cohort, electrothermal atomic absorption spectrometry, GAM, HOME, IQ, LOESS, Rochester, WPPSI-R

## Abstract

**Background:**

Few studies provide data directly relevant to the question of whether blood lead concentrations < 10 μg/dL adversely affect children’s cognitive function.

**Objective:**

We examined the association between blood lead concentrations assessed throughout early childhood and children’s IQ at 6 years of age.

**Methods:**

Children were followed from 6 months to 6 years of age, with determination of blood lead concentrations at 6, 12, 18, and 24 months, and 3, 4, 5, and 6 years of age. At 6 years of age, intelligence was assessed in 194 children using the Wechsler Preschool and Primary Scale of Intelligence–Revised. We used general linear and semiparametic models to estimate and test the association between blood lead concentration and IQ.

**Results:**

After adjustment for maternal IQ, HOME scale scores, and other potential confounding factors, lifetime average blood lead concentration (mean = 7.2 μg/dL; median = 6.2 μg/dL) was inversely associated with Full-Scale IQ (*p* = 0.006) and Performance IQ scores (*p* = 0.002). Compared with children who had lifetime average blood lead concentrations < 5 μg/dL, children with lifetime average concentrations between 5 and 9.9 μg/dL scored 4.9 points lower on Full-Scale IQ (91.3 vs. 86.4, *p* = 0.03). Nonlinear modeling of the peak blood lead concentration revealed an inverse association (*p* = 0.003) between peak blood lead levels and Full-Scale IQ down to 2.1 μg/dL, the lowest observed peak blood lead concentration in our study.

**Conclusions:**

Evidence from this cohort indicates that children’s intellectual functioning at 6 years of age is impaired by blood lead concentrations well below 10 μg/dL, the Centers for Disease Control and Prevention definition of an elevated blood lead level.

Cohort studies of children during the 1980s in North America, Europe, and Australia documented that blood lead concentrations of at least 10 μg/dL are inversely associated with cognitive test scores in children (Needleman and Gatsonis 1990; Pocock et al. 1994; Schwartz 1994). These findings led to the 1991 revision of the Centers for Disease Control and Prevention’s (CDC) definition of an elevated blood lead concentration, which was lowered from 25 to 10 μg/dL (CDC 1991).

Accumulating evidence since 1991 suggests that children’s intellectual ability is adversely affected at blood lead concentrations < 10 μg/dL (Bellinger and Needleman 2003; Canfield et al. 2003a, 2004; Chiodo et al. 2004; Lanphear et al. 2000, 2005; Schnaas et al. 2006; Schwartz 1994; Surkan et al. 2007; Tellez-Rojo et al. 2006). To examine some of this evidence in detail, a working group (Weitzman et al. 2004) was convened by the CDC, and the fifth revision of the CDC’s *Preventing Lead Poisoning in Young Children* was issued in 2005 (CDC 2005b). The working group concluded that the “overall weight of evidence supports an inverse association between blood lead levels < 10 μg/dL and the cognitive function of children,” with the caveat that the available data were limited by the small number of “directly relevant cohort studies”—studies that include multiple measures of lead exposure throughout early life and key covariate information to reduce the potential for residual confounding (CDC 2005b). Despite the conclusions reached by the working group, the CDC definition of an elevated blood lead level was not lowered at that time (CDC 2005b).

This report, based on a prospective study that includes eight measures of children’s blood lead concentrations from 6 months to 6 years of age and that includes measures of key potential confounders in the lead–IQ relation, meets the criteria for a study that is directly relevant to assess questions of possible cognitive effects of lead exposure at blood lead concentrations < 10 μg/dL.

## Methods

### Sample selection

Children participating in the current study were born between July 1994 and January 1995 and were recruited at 24–30 months of age from a previous trial of 276 children enrolled first at 6 months of age (Lanphear et al. 1999). Children and their families were eligible for the dust-control trial if they lived in Rochester, New York, had no plans to relocate in the next 3 months, and the children were between 5 and 7 months of age at the time of the baseline visit. For the current study of lead exposure and cognitive functioning, we excluded low birth weight (< 2,500 g) and preterm (< 37 weeks of gestation) infants, two children with Down syndrome, and one child whose primary language was not English, resulting in 242 children eligible for the current study. At 6 years of age, 194 children (80%) participated; children and parents not participating either moved or could not be located, declined participation or repeatedly missed appointments, or the child had died before this assessment. The Institutional Review Board at the University of Rochester Medical Center approved the study, and all parents or guardians provided written informed consent.

### Collection and analysis of blood samples

Venous blood samples were collected when children were 6, 12, 18, and 24 months of age during the dust-control study, and at 36, 48, 60, and 72 months of age during the current study of cognitive functioning. All collection tubes and needles that were used for specimen collection were provided by the analyzing laboratory, where they were pre-checked by lot number to ensure the absence of any background lead contamination (i.e., < 0.5 μg/dL). All analytical measurements for blood lead were carried out in the Wadsworth Center’s Lead Poisoning and Trace Elements Laboratory (Albany, NY), using a well-established method based on electrothermal atomic absorption spectrometry (ETAAS) (Parsons and Slavin 1993). The Wadsworth Center’s Lead Poisoning and Trace Elements Laboratory is the New York State reference laboratory for this assay and is responsible for operating the New York State Proficiency Testing Program for Blood Lead. It is also a reference laboratory for the blood lead proficiency testing programs operated by the states of Wisconsin and Pennsylvania.

The analytic procedure for lead determination was as follows: Whole blood was diluted 1:9 with phosphate modifier, and a 12-μL aliquot was injected into a Model 4100ZL atomic absorption spectrometer equipped with a transverse-heated graphite atomizer (THGA) and a longitudinal Zeeman-effect background correction system (PerkinElmer Life and Analytical Sciences, Shelton, CT). The THGA instrument was calibrated daily before each run with aqueous lead standards traceable to the National Institute of Standards and Technology (NIST, Gaithersburg, MD). Three concentrations of New York State Department of Health (Albany, NY) blood-based reference materials (including one < 10 μg/dL) were analyzed before, during, and after each analytical run as part of the laboratory’s internal quality assurance program (Parsons et al. 2001). Additional quality assurance validation was obtained through periodic analysis of NIST Standard Reference Material 955a/b Lead in Bovine Blood. The ETAAS analytical method has also been cross-validated against a method based on inductively coupled plasma mass spectrometry (Palmer et al. 2006). All specimens were analyzed in duplicate (independent aliquots), with three furnace injections per analysis. An average lead concentration was calculated across injections for each aliquot by the spectrometer, and the two aliquot means were averaged to derive the lead concentration used in analyses. The method detection limit is estimated at 1.0 μg/dL and the limit of quantitation is approximately 3 μg/dL, based on the International Union of Pure and Applied Chemistry harmonized definitions. Repeatability—the day-to-day precision expressed as a standard deviation—ranged from 0.1 to 0.3 μg/dL at blood lead concentrations < 10 μg/dL based on duplicate measurements over 5 days, whereas it was < 2% above 20 μg/dL. Child’s iron status at 6 years of age was measured by serum transferrin saturation at Rochester General Hospital laboratories.

### Assessment of intelligence

Children were administered the Wechsler Preschool and Primary Scale of Intelligence, Revised, (WPPSI-R) during their 6-year visit at the Rochester General Hospital in Rochester by an examiner trained in pediatric neurobehavioral testing (Brandt and van Gorp 1999). The WPPSI-R was chosen because it provides a thorough sampling of abilities for children with lower than average IQ test scores (Sattler 2001). Children were administered five subtests requiring visual–spatial skills (Object Assembly, Geometric Design, Block Design, Mazes, and Picture Completion) and five subtests requiring verbal skills (Information, Comprehension, Arithmetic, Vocabulary, and Similarities). Combining the scores for all 10 subtests yields the Full-Scale IQ, a global measure of intelligence. Combining the scores for the five visual–spatial subtests yields a Performance IQ; the five verbal subtests yield a Verbal IQ. Ninety-two percent of children with complete data were tested between 72 and 75 months of age (range, 72–80 months), and 156 children (90%) completed all 10 Performance and Verbal IQ subtests. We calculated prorated IQ scores for the remaining 18 children who completed 8 or 9 of the 10 subtests (Sattler 2001; Wechsler 1989). The same examiner conducted all assessments and was unaware of each child’s blood lead concentration.

### Blood lead measures

We constructed four exposure variables from the eight blood lead measures: *a*) lifetime average blood lead concentration, computed by dividing the total area under each child’s age-by-blood-lead curve by 66 (72 months – 6 months); *b*) concurrent blood lead concentration, the blood lead concentration measured on the day of cognitive testing at 6 years of age; *c*) infancy average blood lead concentration (area under the child’s age-by-blood-lead curve from 6 to 24 months); and *d*) peak blood lead concentration, the child’s highest measured blood lead concentration from 6 months through 6 years of age. We used conditional means regression to impute 131 missing age-specific blood lead measures (9% of a total of 1,392) before construction of the lead exposure variables.

### Covariate measures

At each semiannual visit, a parent or guardian was interviewed to obtain information about their child’s medical history and demographic information about the respondent, child, and his or her family. Birth records provided data on perinatal factors including parity, birth weight, and gestational age at birth. The Home Observation for Measurement of the Environment Inventory (HOME) (Caldwell and Bradley 1984) was administered in the child’s home when the child was 24 months of age, and the HOME-Short Form (HOME-SF) (Center for Human Resource Research 1992) was completed by the child’s parent or guardian during the interview at 6 years of age. The HOME-SF has been used with minor modifications in large-scale U.S. longitudinal assessments with good concurrent and predictive validity of vocabulary and achievement test scores (Baker et al. 1993; Bradley et al. 2001). Maternal IQ was assessed during the child’s visit at 3 years of age using the Stanford-Binet IV screening battery (Thorndike et al. 1986).

### Statistical analyses

#### Linear analyses

We estimated the association between each lead exposure measure (lifetime average, concurrent, infancy average, and peak) and each WPPSI-R IQ score (Full-Scale, Performance, and Verbal IQ). Blood lead was modeled categorically to reduce the influence of outlying blood lead values and to demonstrate differences in mean IQ across blood lead groups. Categories were defined as < 5 μg/dL (reference), 5.0–9.9 μg/dL, and ≥ 10 μg/dL for lifetime average, concurrent, and infancy average blood lead concentration. Because of the greater range of values for peak blood lead, concentrations ≥ 10 μg/dL were divided further into two categories: 10.0–14.9 μg/dL and ≥15.0 μg/dL. These natural categories were chosen for their potential relevance to decision making in clinical and health policy settings and to ensure adequate numbers of subjects in each category. We pre-specified a general linear model that included the same predictors of child IQ that had been selected *a priori* and used in a previous report from this cohort (Canfield et al. 2003a; Jusko et al. 2005). In addition to blood lead as a classification factor, the regression model included classification factors for yearly family income measured when the child was 6 years of age (< $10,000, $10,000–24,999, $25,000–50,999, or ≥ $51,000); child’s sex; mother’s highest reported level of education during the 66-month follow-up (< 12 years, 12 years, or > 12 years), race (self-identified as white or nonwhite), and prenatal smoking (yes/no); and covariates birth weight, transferrin saturation, mother’s IQ, and the HOME-SF total score at 6 years of age. The same model was used for all analyses regardless of the dependent variable. Prespecified contrasts for differences in adjacent blood lead groups were estimated and tested to describe the incremental change in IQ across blood lead categories. We also specified a model identical to the primary model except that the categorized blood lead variable was regarded as quantitative. The 1-degree-of-freedom test of this variable can be regarded as a test of trend and is presented for each of the 12 blood lead–IQ combinations (4 blood lead measures × 3 IQ measures). Statistical analyses were conducted using SAS software (version 9.1; SAS Institute Inc., Cary, NC), and all statistical tests were two-sided, with a *p*-value < 0.05 indicating statistical significance.

#### Nonlinear analysis

Because of our previous research indicating a nonlinear dose–response relation and confirmation of this in the analyses in which lead measures are modeled categorically, we conducted a secondary analysis of peak blood lead levels in relation to Full-Scale IQ. This analysis also makes full use of the quantitative nature of the measured lead concentrations. We modeled peak blood lead as the exposure of interest because analysis of this variable helps answer the public health question of setting a maximum allowable blood lead concentration for developing children.

We estimated the dose–response relation using a generalized additive model (GAM), employing a locally weighted scatterplot smooth (LOESS) on the quantitative peak blood lead variable. This model was implemented in SAS version 9.1 (SAS Institute Inc.) using the GAM procedure, specifying a LOESS smoother with 2 degrees of freedom. This semi-parametic GAM model allowed us to adjust parametrically for the same covariates used in the linear analyses and at the same time estimate the association between peak blood lead concentrations and IQ nonparametically. We truncated the top 3% of peak blood lead values (five values between 33.6 and 45.7 μg/dL) to ensure that the shape of the dose–response relation was not influenced by outlying values.

## Results

### Sample characteristics

Of the 194 children and families participating when the child was 6 years of age, 174 had complete information on all explanatory variables and are included in the results described below. Table 1 compares characteristics of children and their families with complete data (*n* = 174), those with missing covariate information (*n* = 20), and those not participating at 6 years (*n* = 48). Except for maternal IQ, characteristics among the three groups were similar.

### Blood lead concentrations

**Figure 1:**
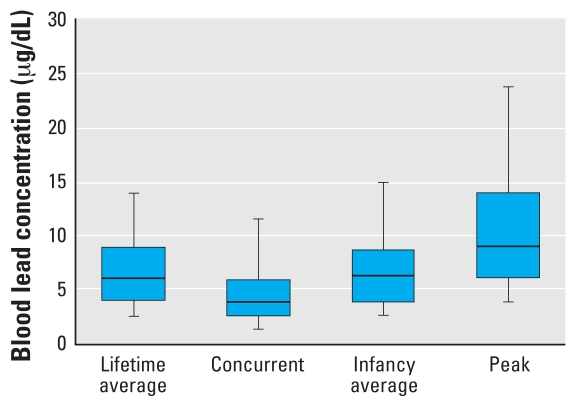
Distributions of blood lead concentrations (*n* = 174). In each box plot, the median value is indicated by the center horizontal line and the 25th and 75th percentiles are indicated by the lower and upper horizontal lines, respectively. The vertical lines represent the 5th and 95th percentiles.

Distributions of each blood lead measure are given in Figure 1. The figure indicates that for no fewer than 75% of children, the lifetime average, concurrent, and infancy average blood lead measures were < 10 μg/dL, and the median blood lead concentration for all lead exposure variables was < 10 μg/dL. Specifically, lifetime average blood lead had a mean of 7.2 μg/dL (median, 6.2 μg/dL; range, 1.4–27.1 μg/dL), with 77% of children averaging < 10 μg/dL through 6 years of age. At the 6-year assessment, concurrent blood lead concentrations averaged 5.0 μg/dL (median, 4.0 μg/dL; range, 1.1–23.7 μg/dL) and 92% of children had measured blood lead concentrations < 10 μg/dL. Infancy average blood lead had a mean of 7.1 μg/dL (median, 6.5 μg/dL; range, 0.7–28.7 μg/dL), with 81% of children averaging < 10 μg/dL for that period. Children’s peak blood lead concentration averaged 11.4 μg/dL (median, 9.4 μg/dL), and ranged from 2.1 to 45.7 μg/dL. Fifty-five percent of children never had a measured blood lead concentration ≥ 10 μg/dL from 6 to 72 months of age.

### Intelligence test results

The mean (± SD) Full-Scale IQ score at 6 years of age was 85 ± 14 (range, 55–146), consistent with previous IQ assessments in this cohort (Canfield et al. 2003a). Full-Scale IQ scores at 6 years of age were correlated with maternal IQ scores (*r* = 0.52, *p* < 0.001), and with the children’s own scores on the Stanford-Binet IV, previously administered at 3 and 5 years of age (Canfield et al. 2003a) (*r* = 0.74, *p* < 0.001; and *r* = 0.82, *p* < 0.001, respectively), at magnitudes consistent with the standardization samples for these instruments (Sattler 2001).

### Blood lead concentrations and IQ

#### Lifetime average blood lead concentration

**Figure 2:**
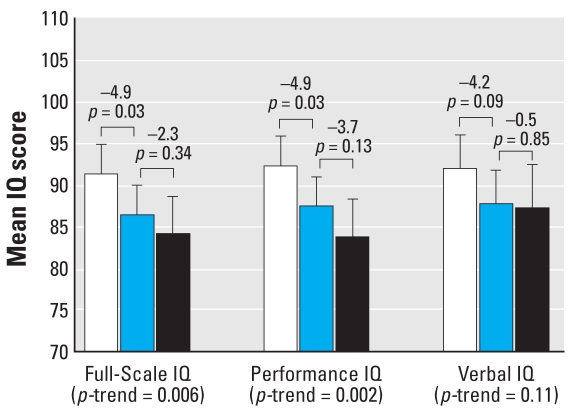
Differences in Full-Scale, Performance, and Verbal IQ associated with increasing lifetime average blood lead concentrations (*n* = 174). Mean IQ levels are adjusted for child’s sex, birth weight, and transferrin saturation; mother’s race, IQ, and education level; HOME-SF total score, family income, and maternal prenatal smoking. Error bars represent 95% confidence intervals. White bars represent the mean IQ of children with blood lead concentrations < 5 μg/dL (*n* = 64), the blue bars represent the mean IQ of children with blood lead concentrations 5–9.9 μg/dL (*n* = 70), and the black bars represent the mean IQ of children with blood lead concentrations ≥ 10 μg/dL (*n* = 40). Values above the brackets represent the mean difference in IQ for adjacent groups and associated *p*-values.

After covariate adjustment, lifetime average blood lead concentration was inversely associated with Full-Scale (*p* = 0.006 for trend) and Performance IQ (*p* = 0.002 for trend) and marginally associated with Verbal IQ (*p* = 0.11 for trend) (Figure 2). Compared with children who had lifetime average blood lead concentrations < 5 μg/dL, children with lifetime average blood lead concentrations between 5 and 9.9 μg/dL scored 4.9 points lower on Full-Scale IQ (91.3 vs. 86.4, *p* = 0.03) and 4.9 points lower on Performance IQ (92.3 vs. 87.4, *p* = 0.03) (Figure 2). Mean Full-Scale IQ scores were 2.3 points lower among children with lifetime average blood lead concentrations ≥ 10 μg/dL than children with lifetime average blood lead concentrations between 5 and 9.9 μg/dL, but this difference was not significant (86.4 vs. 84.1, *p* = 0.34). A similar pattern was noted for Performance IQ (87.4 vs. 83.7, *p* = 0.13).

#### Concurrent blood lead concentration

**Figure 3:**
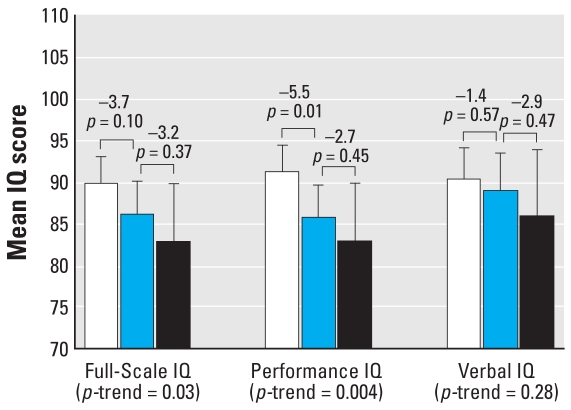
Differences in Full-Scale, Performance, and Verbal IQ associated with increasing concurrent blood lead concentrations (*n* = 174). Mean IQ levels are adjusted for child’s sex, birth weight, and transferrin saturation; mother’s race, IQ, and education level; HOME-SF total score, family income, and maternal prenatal smoking. Error bars represent 95% confidence intervals. White bars represent the mean IQ of children with blood lead concentrations < 5 μg/dL (*n* = 107), the blue bars represent the mean IQ of children with blood lead concentrations 5–9.9 μg/dL (*n* = 53), and the black bar represent the mean IQ of children with blood lead concentrations ≥ 10 μg/dL (*n* = 14). Values above the brackets represent the mean difference in IQ for adjacent groups and associated *p*-values.

A dose–response relation also was observed between concurrent blood lead concentrations and Full-Scale and Performance IQ (*p* = 0.03 and *p* = 0.004 for trend, respectively), but not with Verbal IQ (*p* = 0.28 for trend) after adjustment (Figure 3). The estimated Full-Scale IQ for children with concurrent blood lead concentrations between 5 and 9.9 μg/dL was 3.7 points lower than for children with concurrent blood lead concentrations < 5 μg/dL (89.6 vs. 85.9, *p* = 0.10), and 3.2 points higher than estimated for children with concurrent blood lead concentrations ≥ 10 μg/dL (85.9 vs. 82.7, *p* = 0.37). For Performance IQ, children with concurrent blood lead concentrations between 5 and 9.9 μg/dL scored an average of 5.5 points lower than children with concurrent blood lead concentrations < 5 μg/dL (91.0 vs. 85.4, *p* = 0.01), but the estimated Performance IQ for children with concurrent blood lead concentrations ≥ 10 μg/dL was only 2.7 points lower than children with concurrent blood lead concentrations between 5 and 9.9 μg/dL (85.4 vs. 82.7, *p* = 0.45).

#### Infancy average blood lead concentration

**Figure 4:**
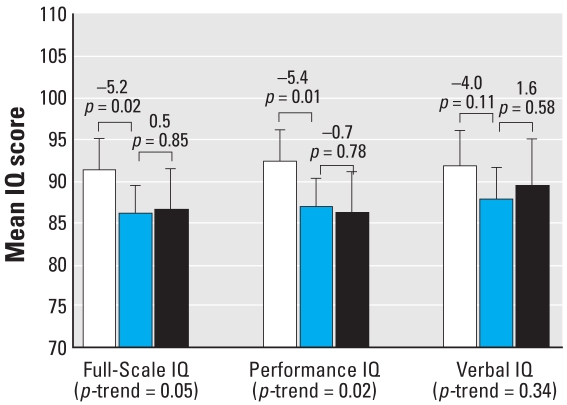
Differences in Full-Scale, Performance, and Verbal IQ associated with increasing infancy average blood lead concentrations (*n* = 174). Mean IQ levels are adjusted for child’s sex, birth weight, and transferrin saturation; mother’s race, IQ, and education level; HOME-SF total score, family income, and maternal prenatal smoking. Error bars represent 95% confidence intervals. White bars represent the mean IQ of children with blood lead concentrations < 5 μg/dL (*n* = 62), the blue bars represent the mean IQ of children with blood lead concentrations 5–9.9 μg/dL (*n* = 79), and the black bars represent the mean IQ of children with blood lead concentrations ≥ 10 μg/dL (*n* = 33). Values above the brackets represent the mean difference in IQ for adjacent groups and associated *p*-values.

Adjusted Full-Scale and Performance IQ scores were associated with infancy average blood lead concentrations (*p* = 0.05 and 0.02 for trend, respectively) (Figure 4). However, there was no significant association of Verbal IQ with infancy average blood lead (*p* = 0.34 for trend). Consistent with results from the lifetime average and concurrent blood lead measures, a dose–response function was observed, with larger Full-Scale and Performance IQ decrements occurring between blood lead categories < 5 μg/dL and 5–9.9 μg/dL than between blood lead categories 5–9.9 μg/dL and ≥ 10 μg/dL (Figure 4). Notably, children with infancy average blood lead concentrations between 5 and 9.9 μg/dL scored 5.2 points lower on Full-Scale IQ (91.1 vs. 85.9, *p* = 0.02) and 5.4 points lower on Performance IQ (92.2 vs. 86.7, *p* = 0.01) than did children with infancy average blood lead concentrations < 5 μg/dL.

#### Peak blood lead concentrations

**Figure 5:**
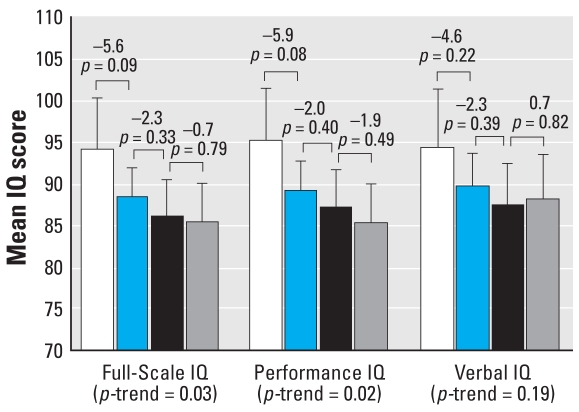
Differences in Full-Scale, Performance, and Verbal IQ associated with increasing peak blood lead concentrations (*n* = 174). Mean IQ levels are adjusted for child’s sex, birth weight, and transferrin saturation; mother’s race, IQ, and education level; HOME-SF total score, family income, and maternal prenatal smoking. Error bars represent 95% confidence intervals. The bars represent the mean IQ of children with blood lead concentrations < 5 μg/dL (*n* = 17; white); 5–9.9 μg/dL (*n* = 79; blue); 10–14.9 μg/dL (*n* = 41; black); and ≥ 15 μg/dL (*n* = 37; gray). Values above the brackets represent the mean difference in IQ for adjacent groups and associated *p*-values.

Both Full-Scale and Performance IQ exhibited a dose–response relation with peak blood lead concentration. Again, lower IQ scores were associated with higher peak blood lead concentrations (*p* = 0.03 and *p* = 0.02 for trend, respectively). Verbal IQ exhibited a less consistent trend with peak blood lead concentration (*p* = 0.19 for trend) (Figure 5). Comparing estimated Full-Scale IQ across the four peak blood lead categories, the difference between blood lead category 1 and 2 was 5.6 IQ points (93.9 vs. 88.3, *p* = 0.09), but only a 2.3-point IQ difference was observed comparing groups 2 and 3 (88.3 vs. 85.9, *p* = 0.33), and an even smaller difference was observed comparing groups 3 and 4 (85.9 vs. 85.2, *p* = 0.79). A similar pattern was observed for Performance IQ.

#### Peak blood lead concentration and IQ: Nonlinear function

**Figure 6:**
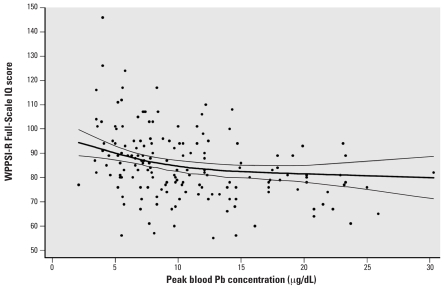
Full-Scale IQ as a function of peak blood lead concentration from 6 months to 6 years (*n* = 169), with 95% confidence intervals. The individual points represent the unadjusted peak blood lead concentrations and Full-Scale WPPSI-R IQ scores.

A plot of the nonlinear relation between peak blood lead and Full-Scale IQ is shown in Figure 6. An inverse association (*p* = 0.003) between the child’s maximum (peak) blood lead concentration and Full-Scale IQ was apparent down to 2.1 μg/dL, the lowest measured peak concentration in our sample. Further, the slope of the blood lead–IQ relation was steeper at lower than at higher levels of exposure. For instance, IQ decreased by approximately 1.2, 0.32, and 0.15 points per 1-μg/dL increase in peak blood lead over the range of 2.1–10 μg/dL, 10–20 μg/dL, and 20–30 μg/dL, respectively.

## Discussion

The findings of this study are directly relevant to the question of whether blood lead concentrations < 10 μg/dL adversely affect children’s cognitive functioning: Blood lead was measured on up to eight occasions during infancy and early childhood; the lifetime average blood lead concentration was 7.2 μg/dL, and more than half of the children never had a measured blood lead concentration of ≥ 10 μg/dL; we gathered extensive information about influences other than lead exposure that are known to affect intellectual development; and we assessed intelligence at an age when IQ is measured reliably and is a strong predictor of intelligence during adolescence and adulthood. The results show that childhood blood lead concentrations are inversely related to IQ scores, whether lead exposure is measured by lifetime and infancy average measures, maximal (peak) exposure, or on the same day the IQ test is administered. This pattern of findings is most apparent for the Full-Scale and the Performance IQ scores. In particular, children with blood lead concentrations in the 5–9.9 μg/dL range had significantly lower IQ scores than children who had blood lead concentrations < 5 μg/dL. Further, additional nonlinear analysis of peak exposure throughout early childhood indicated that blood lead levels as low as about 2 μg/dL may be associated with declines in Full-Scale IQ. These findings also add to the body of evidence that the effect of blood lead on child intellectual development is larger for equal increments of lead < 10 μg/dL than it is at higher levels.

The analytic approach in this study allowed for direct comparisons between children with blood lead concentrations < 5 μg/dL with those who had levels > 5 μg/dL but still below the CDC definition of an elevated blood lead level (i.e., 5–9.9 μg/dL). The declines in IQ observed with this approach reinforce the conclusions of previous findings from this cohort (Canfield et al. 2003a, 2003b, 2004; Lanphear et al. 2005) that children are adversely affected by blood lead concentrations < 10 μg/dL. Findings from the current investigation also extend the previous findings by demonstrating that the low-level associations reported at 3 and 5 years of age are not specific to a particular IQ test. Whereas the Stanford-Binet IV test was administered at 3 and 5 years of age, the WPPSI-R was used in the current investigation. Moreover, these results also indicate that the potentially adverse cognitive effects of blood lead concentrations < 10 μg/dL persist to 6 years of age—an age when IQ is measured more reliably and is a stronger predictor of future achievement than when measured at earlier ages.

A second pattern in our data is that Performance IQ is more strongly associated with blood lead levels than is Verbal IQ. This result is consistent with the findings from other cohort studies. In particular, considering the 15 relevant cognitive assessments of children from 3–13 years of age in these studies, 11 find blood lead levels associated with poorer performance on Performance IQ or related tests of visual–spatial or visual–motor functioning (Bellinger et al. 1991; Dietrich et al. 1991, 1992, 1993; Factor-Litvak et al. 1999; McMichael et al. 1988; Stiles and Bellinger 1993; Tong et al. 1996; Wasserman et al. 1997). For three of the four remaining studies, one or more key subtests on the Performance scale (i.e., block design, picture completion, mazes) were significantly associated with children’s blood lead concentrations, although the overall subscale score was not (Baghurst et al. 1992; Stiles and Bellinger 1993; Tong et al. 1996). In one notable exception, no association with lead exposure was found when children from the Boston cohort were examined with a neuropsychological test designed specifically to evaluate visual–perceptual and visual–motor skills in children (Stiles and Bellinger 1993). It appears that verbal abilities become somewhat more sensitive indicators only during middle and later childhood. This runs counter to the fact that tests of verbal abilities tend to show slightly greater test–retest reliability than visual–spatial tests (Sattler 2001).

This study is limited in its ability to describe fully the blood lead–IQ relation at concentrations > 10 μg/dL, and thus the estimated mean IQ for children in the ≥10 μg/dL groups may be imprecise. In addition, because prenatal maternal blood and umbilical cord blood specimens were unavailable, we were unable to assess the potential impact of prenatal exposures. Though recent evidence suggests an association between *in utero* exposures and neurodevelopment (Hu et al. 2006; Schnaas et al. 2006), at least two studies reporting on both pre- and postnatal lead concentrations nevertheless demonstrate that postnatal lead concentrations are associated with adverse neurodevelopmental outcomes, independent of prenatal lead levels (Schnaas et al. 2006; Wasserman et al. 2000).

The observational design of this study makes it necessarily vulnerable to potential misclassification and residual confounding. To reduce the possibility of misclassification of exposure, blood lead was assessed up to eight times during infancy and early childhood. Compared with cross-sectional studies in which blood lead concentrations are assessed at only one time point, multiple lead determinations provide a more complete representation of children’s exposure to lead, particularly during the period of 18–36 months of age when blood lead levels are typically highest and most variable. To reduce the potential for residual confounding (Bellinger 2004a), several additional covariate measures were examined in secondary analyses. In addition to the covariates included in the primary analysis reported here, we also considered breast-feeding, the HOME scale score at 24 months of age, and other measures of the child-rearing environment (crowding in the home, and household income after accounting for additional government subsidies and housing expenses). Some of these covariates were considered as potential confounders instead of or in addition to variables in the *a priori* model, but their inclusion did not change the estimated mean IQs by > 5%. As a further step to reduce the potential for residual confounding, we examined some covariates in polynomial form and by splines. These methods also did not materially affect our results.

The importance of these findings should be evaluated in the context of current levels of lead exposure common in children today. Primarily because of the elimination of lead as an additive to paint and gasoline, blood lead levels among children have declined greatly over the last three decades: The prevalence of an elevated blood lead concentration (≥10 μg/dL) among all children in the United States between 1 and 5 years of age declined from 77.8% in 1976–1980 to just 1.6% in 1999–2002 (CDC 2005a). It can be fairly asked, then: What is the relevance of our finding that lifetime blood lead levels between 5 and 10 μg/dL are associated with a 4.9-point decline in IQ? NHANES (National Health and Nutrition Examination Study) data from 1988–1994 indicate that approximately 26% of children 1–5 years of age had blood lead concentrations between 5 and 10 μg/dL (Bernard and McGeehin 2003). Though this number probably overestimates the prevalence today (because of continuing declines in lead exposure), the proportion of children with blood lead levels of at least 5 μg/dL but < 10 μg/dL in some economic, ethnic minority, and geographic subpopulations is likely to be much greater (Bernard and McGeehin 2003). For example, between 1988 and 1994, 1- to 5-year-old children living below the NHANES poverty income ratio were 60% more likely to have a blood lead concentration between 5 and 10 μg/dL compared with children living above the poverty income ratio. From those same data, Bernard and McGeehin (2003) estimated that non-Hispanic black and Mexican-American children were 3.3 and 2.4 times more likely to have a blood lead concentration between 5 and 10 μg/dL, compared with non-Hispanic white children In addition, 1- to 5-year-olds living in the northeast were 5.8 times more likely to have a blood lead concentration between 5 and 10 μg/dL compared with children living in the western United States (Bernard and McGeehin 2003).

Important decisions about school placement, aptitude for college work, and opportunities for training and advancement in the workplace are often based on an individual’s performance in relation to arbitrary cutoff scores on IQ-like tests (Bellinger 2004b). Thus, a small decline in an IQ-like score can have a profound impact for individuals that earn scores slightly below an arbitrary cutoff. Indeed, a difference of only a few points on an aptitude test prevents many otherwise eligible students from having an opportunity to pursue higher education. As has been noted by others (Bellinger 2004b; Gilbert and Weiss 2006; Needleman et al. 1982), the importance of a small decline in IQ also can be gauged by taking a societal perspective. For example, a five-point downward shift in IQ results in a disproportionate (57%) increase in the number of children in a population with IQ scores in the extremely low range (< 70) (Gilbert and Weiss 2006). An IQ test score of 70 is a commonly used criterion for designating a child as having mild mental retardation and is a major consideration in whether or not a child should be placed in a special education program, resulting in an approximate doubling of the cost for his or her education. Similarly, a five-point shift in the average IQ of a population would lead to a 40% reduction in the number of children who score in the very superior range (IQ > 130). An IQ score of 130 is often a requirement for access to public school–based programs for gifted and talented children (Winner 1997). Thus, when viewed from the perspective of the individual and society as a whole, a small effect of lead on IQ can be very costly. The current study estimates that effects of this magnitude may be caused by an increase in blood lead concentrations from < 5 up to 10 μg/dL.

## Figures and Tables

**Table 1:** Characteristics of children, mothers, and families when the child was 6 years of age.

Characteristic	Children with complete data (*n* = 174)	Children with incomplete data (*n* = 20)^a^	Not participating at 6 years (*n* = 48)
Children			
Female sex (%)	50	45	62
Birth weight (g)	3,301 ± 422	3,460 ± 439	3,293 ± 497
Weeks of gestation	39.5 ± 1.2	39.8 ± 0.7	39.5 ± 1.3
Full-scale IQ	85.4 ± 14.4	84.8 ± 12.6	—
Lifetime average blood lead (μg/dL)	7.2 ± 4.1	6.2 ± 4.0	6.6 ± 2.8
Concurrent blood lead (μg/dL)	5.0 ± 3.3	4.1 ± 3.1	—
Infancy average (μg/dL)	7.1 ± 3.9	6.8 ± 4.0	6.8 ± 3.8
Peak blood lead (μg/dL)	11.4 ± 7.3	11.4 ± 9.1	10.2 ± 5.7
Transferrin saturation (%)	20.7 ± 8.6	16.1 ± 6.3	—
Mothers			
Age at delivery (years)	24.8 ± 6.6	25.2 ± 5.5	24.4 ± 5.3
Number of prenatal visits	11.2 ± 4.2	10.5 ± 4.4	9.9 ± 3.4
Smoked during pregnancy (%)	24	26	26
Nonwhite race (%)	74	50	67
IQ*	81.6 ± 12.6	93.9 ± 12.5	84.2 ± 9.8
Education (years)	12.3 ± 1.9	12.3 ± 2.0	—
Household income [US$ (%)]			
< 10,000	28	28	—
10,000 – 24,999	45	22	—
25,000 – 50,999	21	28	—
≥51,000	6	22	—
HOME-SF total score	11.3 ± 2.5	11.7 ± 2.7	—

Data are presented as mean ± SD unless otherwise indicated. Mean differences across groups tested with chi-square, analysis of variance, and Kruskal–Wallis tests respectively, for categorical, interval, and ordinal variables.

a Data missing for some characteristics and some study participants.

* *p* < 0.05 for comparison between children with complete data (*n* = 174) and children with incomplete data (*n* = 20).
